# Therapeutic effect of intramedullary reaming and nailing for long bones lengthening in children with Ollier disease and Maffucci syndrome on enchondromas: multicentric retrospective case series

**DOI:** 10.1051/sicotj/2024035

**Published:** 2024-10-25

**Authors:** Soline Bonneau, Samuel Georges, Bernard Fraisse, Edouard Haumont, Yan Lefèvre, Nicolas Bremond, Zagorka Pejin, Philippe Violas

**Affiliations:** 1 Service de Chirurgie Pédiatrique, CHU Angers 4 Rue Larrey 49100 Angers France; 2 Service de Chirurgie orthopédique et traumatologie pédiatrique, Hôpital Necker Enfants malades – AP-HP 149 Rue de Sèvres 75015 Paris France; 3 Service de Chirurgie Pédiatrique, CHU Rennes, Hôpital Sud 16 Boulevard de Bulgarie 35200 Rennes France; 4 Service de Chirurgie Orthopédique et Traumatologique de l’enfant et de l’adolescent, Hôpital des Enfants – Groupe hospitalier Pellegrin, CHU de Bordeaux Place Amélie Raba Léon 33000 Bordeaux France; 5 Service de Chirurgie Pédiatrique, Chirurgie orthopédique et traumatologique, CHU Grenoble Alpes Boulevard de la Chantourne 38700 La Tronche France

**Keywords:** Lengthening nail, Maffucci syndrome, Ollier’s syndrome, Enchondromas, Leg length discrepancy

## Abstract

*Introduction*: Leg length discrepancy (LLD) and malalignment of long bones are frequent orthopedic problems encountered in Maffucci syndrome and Ollier disease (OD). Orthopedic surgeons used historically external fixators to address the deformities. In this multicentric case series, we propose the use of motorized intramedullary nails. *Methods*: We retrospectively reviewed for 9 years, in four different centers, patients with OD and Maffucci’s syndrome that had lengthening nails for LLD with or without associated deformities. The minimum follow-up period was 24 months. We reported complications, clinical tolerance of lengthening, lengthening rate and target, bone healing index, and EQ-5D-Y functional and visual analog scores (VAS). We also saw on X-rays the whole lengthened bone and its regenerate zone to assess the evolution of the enchondromas. *Results*: we used the nailing technique in 8 femurs and 2 tibias in 8 patients (mean age: 13.3 years, range: 11–16, mean follow-up time: 53.8 months, range: 26–108). The mean correction amount was 6.44 cm for the femur over 76.8 days and 3.75 cm over 44 days for the tibia with a mean VAS score of 6.63/15 and mean EQ-5D-Y of 81/100. The lengthening goal was achieved in all patients. No mechanical complications were noted. The medullary canal of the operated bones showed improvement and healing in 8 out of 10 segments. *Discussion*: Besides achieving the goals of surgery with good functional outcomes, lengthening nails has a therapeutic effect on enchondromas with fewer complications than traditional correction methods.

## Introduction

Multiple enchondromatosis (Ollier disease, OD) is an uncommon non-hereditary condition. It is characterized by numerous enchondromas in the bones [1]. Maffucci syndrome is a disorder of enchondromatosis associated with numerous anomalies (hemangiomas) involving the soft tissue [2]. During childhood, enchondromas grow asymmetrically in the metaphysis near the physis of long bones. Therefore, patients often develop bone fragility, limb length discrepancy (LLD), and multiplanar deformities. During adulthood, these benign tumors risk potential transformation into chondrosarcoma [3–7]. The risk is estimated at 25% by the fourth decade of life and up to 40% as lifetime risk [8, 9].

Enchondromas are surgically treated by curettage with or without grafting [10–14]. Surgical procedures for deformities and LLD vary widely. Due to the rarity of this condition and the weakened nature of the affected bones, there is no consensus concerning the best surgical technique and implants. Uniplanar and circular frames with or without intra-medullary nailing gained popularity in achieving satisfactory results [15]. Complications with these procedures are common: joint stiffness, pin track infection, and pathological fractures [16–20]. Moreover, these techniques do not address the treatment of enchondromas. Therefore, an implantable lengthening nail procedure seems a suitable alternative with fewer complications. Very few studies reviewed this technique in multiple enchondromas conditions [21, 22].

We hypothesized that implantable lengthening nails in OD and Maffucci syndrome is a safe technique that corrects LLD, and axial deformity if needed and treats enchondromas lesions when reaming before introducing the nail, exerting a curettage-like effect. Hence, the purpose of this study was to evaluate the outcome of this procedure.

## Materials and methods

Between 2014 and 2023, we reviewed our charts and radiographs in 4 different centers in France on all patients with OD or Maffucci Syndrome, with the diagnosis confirmed with genetic tests. Patients were included in this study if they had undergone limb lengthening procedures using implantable lengthening nails (PRECISE^®^, Nuvasive Inc., San Diego, CA, USA) with a minimal clinical and radiological follow-up period of 24 months. The local investigational review board approved this study, and all the patients’ parents gave written informed consent.

The surgical technique was done according to the previously described lengthening procedures for the tibia and femur [23, 24]. We performed DeBastiani corticotomy in the transition zone between the diaphysis and the metaphysis. If the deformity is present and limb alignment is needed, we completed the correction described by Baumgart [25]. We introduced nails in an antegrade or retrograde fashion way. We distracted 1 mm at the end of the surgery for each patient to evaluate the functioning of the nail. We began lengthening 5–9 days postoperatively at a rate of two times per day. We adjusted the lengthening rate depending on each patient’s clinical tolerance, union rate, and bone quality. No weight bearing was allowed during the lengthening process, followed by partial weight bearing (15–30 kg depending on the nail size and patient’s weight) with two crutches until the bony union. Rehabilitation programs also focused on hip, knee, and ankle range of motion and strengthening muscular exercises 5–6 days per week. Follow-up consisted of radiological and clinical exams every 1–2 weeks until the end of the lengthening, then every 3 months.

We obtained from the charts for each patient his age, sex, date of nail insertion, lengthening rate, lengthening target, clinical tolerance for lengthening, and complications. The lengthening goal was either equalizing the limbs if the amount of LLD is lesser than the maximum ability of the nail (50 mm for the tibial nail and 80 mm for the femoral nail) or achieving this maximal capacity. We analyze the regenerate zone and the lengthened long bone to assess the evolution of the pre-existing enchondromas on all the radiographs obtained. We measured the bone healing index (BHI), which is the amount of time until bony union in days with the nail in place per the amount of lengthening in centimeters (cm). We considered a bony union when we noticed the presence of at least three cortices.

After three months of achieving bony union, we evaluated all patients’ EQ-5D-Y functional and visual analog scores (VAS) [26]. We calculated descriptive statistics using Microsoft Excel^®^.

## Results

We investigated seven patients with OD and one with Maffucci syndrome. They underwent 10 lengthening procedures with implantable lengthening nails (Table 1). There were eight femoral limb segments and two tibias. Four patients had the left limb shorter. One patient had two intermittent femoral retrograde lengthenings with a tibial lengthening (patient 2). There were 3 girls and 5 boys. The mean age was 13.3 years (range: 11–16). The mean follow-up time was 53.8 months (range: 26–108). The mean pre-operative LLD was 8.6 cm (range: 3.5–16.8). The mean pre-operative discrepancy was 8.9 cm for the femur (range: 4.5–16.8) and 3.75 cm for the tibia (range: 3.5–4.0). We corrected seven multiplanar deformities associated with LLD.

**Table 1:** Table resumes patients’ characteristics. M: Male, F: Female, Fe: Femur, T: Tibia, R: right, L: Left, LLD: leg length discrepancy, BHI: Bone Healing Index.

No patient	Age (years)	Sex	Bone lengthened	Side	Syndrome	Associated deformity correction	Pre-op LLD (cm)	Lengthening (cm)	Lengthening/initial bone length (%)	Post-op LLD (cm)	Time for bony union (days)	BHI	Complications	Hardware removal
1	15	M	F	R	Ollier’s disease	None	6.6	6	14	0.6	120	19.7	–	No
2.1	11	M	F	L	Ollier’s disease	Knee recurvatum	8	5	14	3	120	24	–	Yes
2.2	14	M	T	L	Ollier’s disease	Knee valgus + Recurvatum	4	4	13	0	150	37.5	–	
2.3	16	M	F	L	Ollier’s disease	Knee varus + Flessum	4.5	4	9	0.5	120	26.6	–	
3	12	F	F	R	Ollier’s disease	Coxa valga	16.8	8	24	8.8	210	26.3	–	Yes
4	16	M	T	L	Ollier’s disease	Knee valgus	3.5	3.5	14	0	150	42.8	–	Yes
5	11	F	F	R	Ollier’s disease	Knee valgus + Recurvatum	7	8	27	−1	155	19.4	–	No
6	14	M	F	L	Maffucci’s syndrome	Knee varus + femoral retroversion	13	8	24	5	390	48.8	Non union	Yes
7	13	F	F	L	Ollier’s disease	None	9.1	7.5	22	1.6	195	26	–	No
8	11	M	F	R	Ollier’s disease	Knee varus	4.7	5	14	−0.3	125	25	–	Yes

The mean amount of correction was 6.44 cm for the femur (range: 4–8) over a mean duration of 76.8 days (range: 53–100) and 3.75 cm for the tibia over 44 days (range: 38–50). We obtained the goal of lengthening in all patients. We noted bony union after a mean period of 179.4 days for the femur (range: 120–390) and 150 days for the tibia. The mean BHI was 29.6 days/cm (range: 19.4–48.8), with 27 days/cm for the femur and 40.2 days/cm for the tibia. At three months postoperatively, we evaluated the mean VAS score at 6.63 points/15 (range: 5–8) and the mean EQ-5D-Y at 81/100 (range: 70–95).

**Figure 1:**
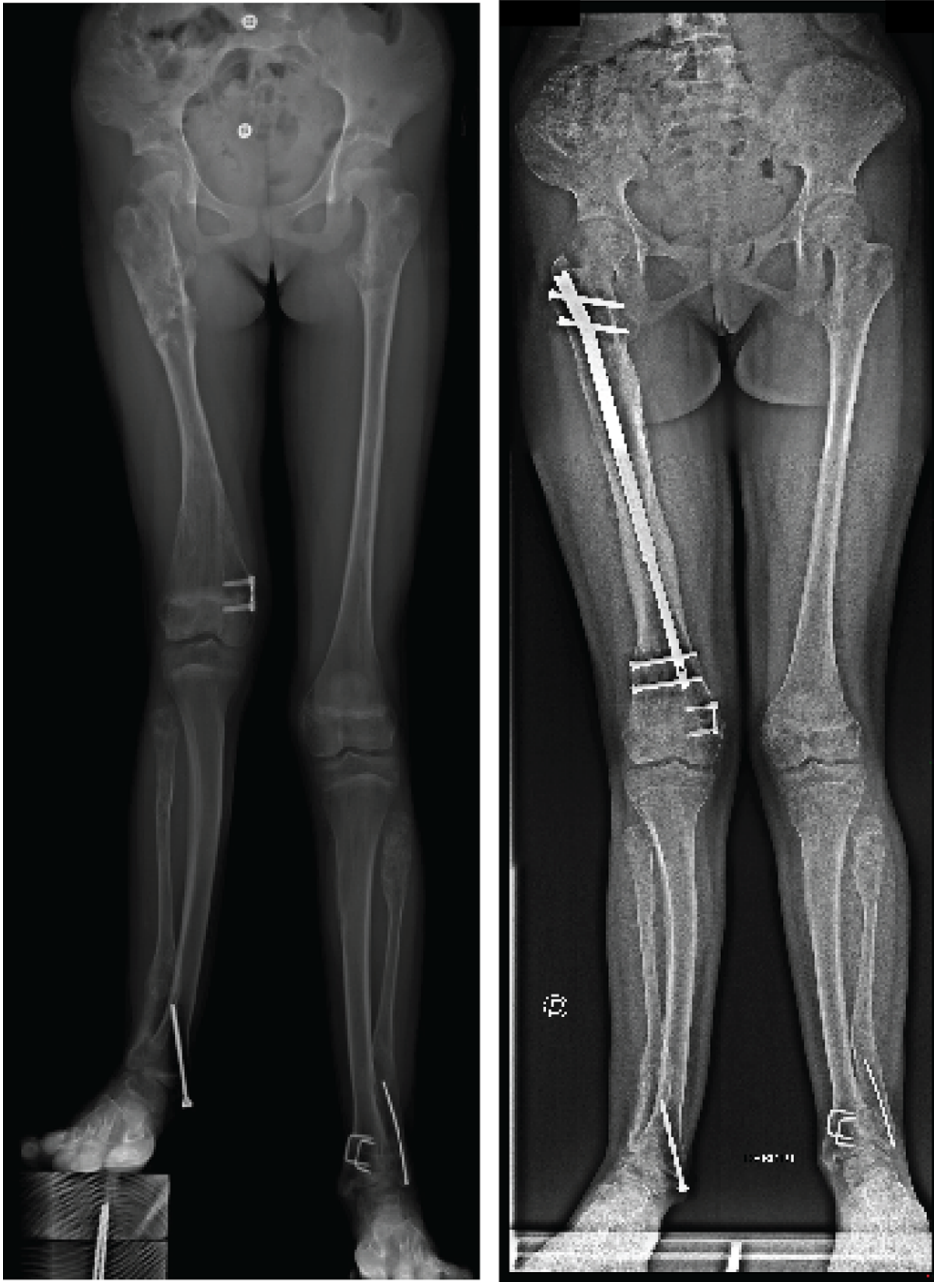
Corresponds to patient No. 5 and shows “Curetage-like effect” on the enchondromas of the reamed area at the end of nail lengthening. (A) Pre-operative; (B) 1 year post-operative.

**Figure 2:**
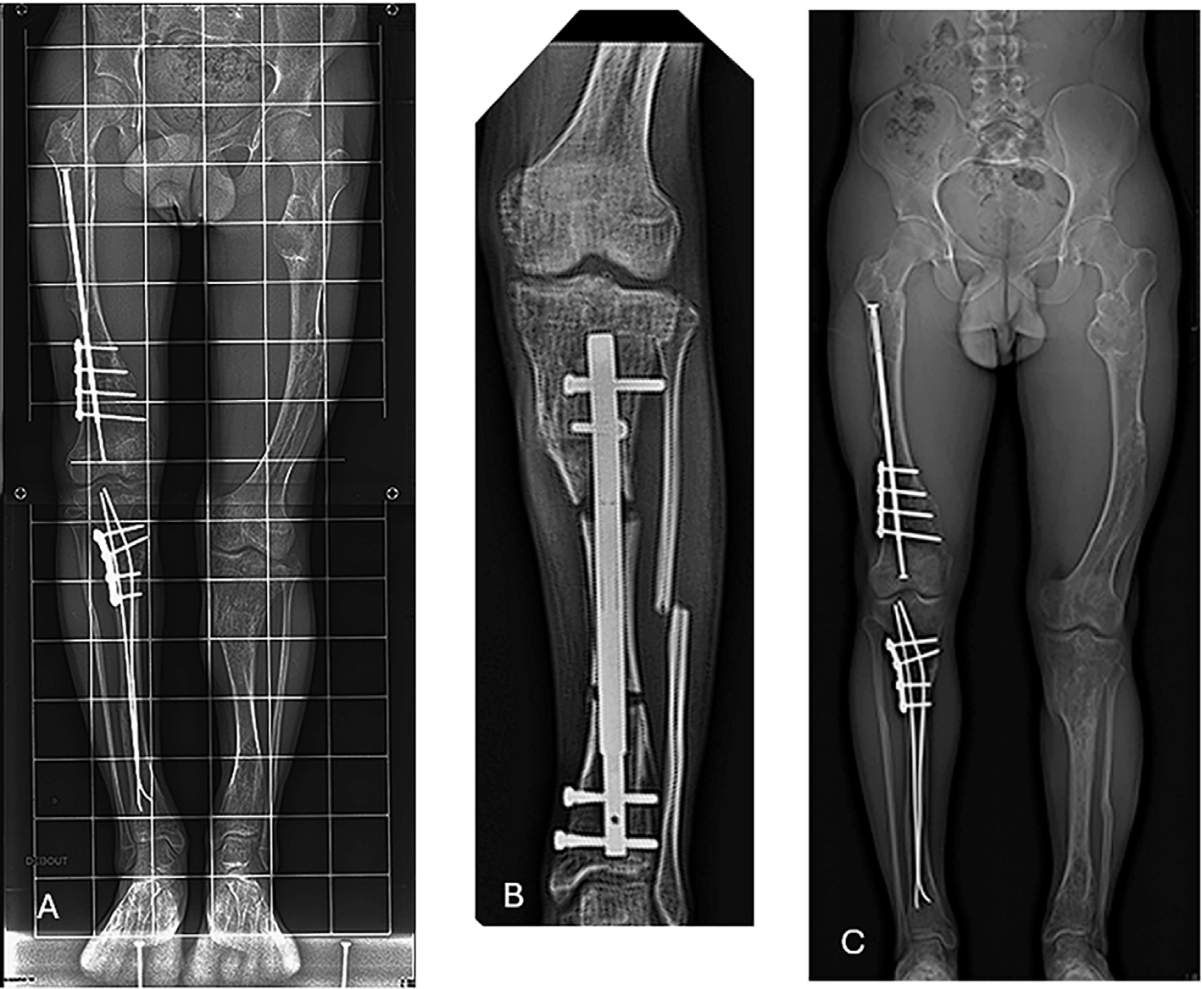
Corresponds to patient No. 4 and shows the effect of reaming in the diaphyseal tibial area after 1 year of removal of hardware. (A) Pre-operative; (B) Post-operative in the OR; (C) Post-operative at 1 year of removal of hardware.

There were six extralesional osteotomies, two in the transitional zone and two in the enchondroma region. We had 14 proximal and 14 distal intralesional screws and 6 proximal and 6 distal extra-lesional screws. When analyzing the radiographs, we found that all the regenerate zones were lesions-free (Figure 1). The reamed areas showed improvement in the cortical and medullary appearance of the bone, with an improvement of the enchondromas in all the lengthened bones (Figure 2).

We had no mechanical complications or nail migration. One patient with Maffucci syndrome presented nonunion. After 4 months of the end of femoral lengthening (patient 6). We first addressed it with the dynamization effect by cyclic compression/distraction (1 mm/day) for 60 days with no improvement. He had a tibial allograft revision surgery 7 months postoperatively and achieved bony union 4 months later. We removed six nails after a mean period of 29 months (range: 22–48). We did not report any later pathological fracture.

## Discussion

Few papers suggest implantable lengthening nails in enchondromatosis [21, 22]. Our findings show that this type of nail is suitable for treating LLD with or without deformities in patients with OD and Maffucci syndrome. It is well tolerated by patients, as shown by our functional and pain scores, and achieves the desired goals of the surgery with very few complications. It also appears to treat enchondromas in the reamed area of the bone.

We have achieved the same leg length and deformity correction in all patients with only one nonunion. In a recent systematic review concerning limb lengthening in OD, Angelini et al. noted that external fixators (monolateral or circular frames) were the most used technique (90% of the procedures), with 29% of complications without taking into consideration mild pin track infections which represent 50% [15]. To compare the outcomes between our series and the available data, we used the BHI as defined by Angelini et al. This BHI was the same as the unique published series using this same technique with the same nail PRECISE^®^ [22]. One significant difference between these two series is the BHI of our two tibias, estimated at 40.15 days/cm compared to 28.8 in theirs. This marked discrepancy between these results comes from the lengthening of our two tibias, which was associated with a significant deformity correction.

We had no complications with the distal and proximal screws, even though most were intra-lesional. We adjusted the lengthening rate to the radiological regenerate zone and clinical tolerance. We had no joint subluxation or dislocation. The only complication in this series was nonunion of the femur in a Maffucci Syndrome that needed revision surgery with tibial allograft. Before its lengthening procedure, the patient had a telescopic nail for an earlier fracture and deformity. We had to remove this nail during the lengthening surgery through the osteotomy site. The periosteal and endosteal devascularization of the femur, with the later lengthening of 8 cm, played a significant role in this nonunion.

Historically, surgical techniques used to re-establish leg length and deformity correction are based on external fixators. This technique causes discomfort for patients, a problematic post-operative period with postponed rehabilitation, less satisfactory outcomes, and considerable complications [15]. As shown by our functional and pain scores, this lengthening nail not only achieves surgery goals but also improves the quality of life for patients during the lengthening period. It is a safe technique that offers the same functional results as an external fixator and, if used correctly, gives patients fewer complications and better quality of life.

Conservative treatment for enchondromatosis does not exist yet. OD and Maffucci syndrome are rare (1/100,000) non-hereditary disorders. Recent studies showed they are caused by a mutation in 2 genes (*PTHR1* and *IDH1*) [27]. We consider surgical treatment of enchondromas in cases of leg length discrepancy, skeletal deformities, fractures, cosmetic and functional impairment, or malignant transformation. This transformation risk is around 20–50% in OD and is more significant in Maffucci’s syndrome, with a much worse prognosis [6]. Hence, it is important to manage enchondromas when necessary and monitor for potential malignancy. The primary rationale for suggesting hardware removal after lengthening and union is achieved. So far, we have extracted 6 out of 9 nails, with plans to remove the remaining three eventually. Surgical treatment consists of a curettage [10]. Reaming the medullary canal before inserting the nail exhibits the same effect of phalangeal curettage on enchondromas. Therefore, with lengthening nails, one can simultaneously achieve the right length and fix deformities while also treating enchondromas during the same process. Furthermore, it would be beneficial to observe the long-term outcomes of reaming and nailing through an extended follow-up into adulthood for patients with enchondromas.

One limitation of this technique is the quantity of lengthening offered by the nail, which is limited to 5 or 8 cm. However, careful examination and follow-up of these patients and choosing to perform a contralateral epiphysiodesis would reduce the significant discrepancy. A further limitation of this study was the lack of anatomopathological samples from the reaming product. Moreover, it is a retrospective study with a limited number of cases of a rare disease. Our findings seem encouraging to use the motorized nail in these patients, but a more extensive prospective series would clarify this.

## Data Availability

The data generated during this study are available on request from corresponding author Samuel Georges.
